# Enhancement of lung tumorigenesis in a *Gprc5a *Knockout mouse by chronic extrinsic airway inflammation

**DOI:** 10.1186/1476-4598-11-4

**Published:** 2012-01-12

**Authors:** Peter Barta, Carolyn Van Pelt, Taoyan Men, Burton F Dickey, Reuben Lotan, Seyed Javad Moghaddam

**Affiliations:** 1 Department of Thoracic/Head and Neck Medical Oncology, The University of Texas MD Anderson Cancer Center, Houston, Texas, USA; 2 Department of Veterinary Medicine and Surgery, The University of Texas MD Anderson Cancer Center, Houston, Texas, USA; 3 Department of Pulmonary Medicine, The University of Texas MD Anderson Cancer Center, Houston, Texas, USA

**Keywords:** lung cancer, inflammation, COPD, Gpcr5a, NTHi

## Abstract

**Background:**

Although cigarette smoking is the principal cause of lung carcinogenesis, chronic obstructive pulmonary disease (COPD), an inflammatory disease of the lung, has been identified as an independent risk factor for lung cancer. Bacterial colonization, particularly with non-typeable *Haemophilus influenzae *(NTHi), has been implicated as a cause of airway inflammation in COPD besides cigarette smoke. Accordingly, we hypothesized that lung cancer promotion may occur in a chronic inflammatory environment in the absence of concurrent carcinogen exposure.

**Results:**

Herein, we investigated the effects of bacterial-induced COPD-like inflammation and tobacco carcinogen-enhanced tumorigenesis/inflammation in the retinoic acid inducible G protein coupled receptor knock out mouse model (Gprc5a-/- mouse) characterized by late-onset, low multiplicity tumor formation. Three-month-old Gprc5a-/- mice received 4 intraperitoneal injections of the tobacco-specific carcinogen, NNK, followed by weekly exposure to aerosolized NTHi lysate for 6 months. The numbers of inflammatory cells in the lungs and levels of several inflammatory mediators were increased in Gprc5a-/- mice treated with NTHi alone, and even more so in mice pretreated with NNK followed by NTHi. The incidence of spontaneous lung lesions in the Gprc5a-/- mice was low, but NTHi exposure led to enhanced development of hyperplastic lesions. Gprc5a-/- mice exposed to NNK alone developed multiple lung tumors, while NTHi exposure increased the number of hyperplastic foci 6-fold and the tumor multiplicity 2-fold. This was associated with increased microvessel density and HIF-1α expression.

**Conclusion:**

We conclude that chronic extrinsic lung inflammation induced by bacteria alone or in combination with NNK enhances lung tumorigenesis in Gprc5a-/- mice.

## Background

Worldwide, lung cancer remains the leading cause of cancer death in both men and women, even though an extensive list of modifiable risk factors has long been identified [1]. Cigarette smoking is the principal cause of lung carcinogenesis [2]. However several studies have found that smokers with chronic obstructive pulmonary disease (COPD) have an increased risk of lung cancer (3 to 10 fold) compared to smokers with comparable cigarette exposure but without COPD [3,4]. It has also been shown that increased lung cancer mortality is associated with a history of COPD, even among persons who had never been active smokers [5].

The pooled global prevalence of COPD in adults 40 years or older is 9%, and it is a leading cause of morbidity and mortality in the United States [6]. Histopathologic studies have clearly demonstrated lung inflammation in COPD [7]. Smoking also causes most cases of COPD [8], however, among smokers with COPD, inflammation persists and lung function continues to deteriorate even following withdrawal of cigarette smoke [8]. These facts suggest that the later phase of lung carcinogenesis may occur in a chronic inflammatory environment in the absence of concurrent carcinogen exposure.

We have previously established a COPD-like mouse model of airway inflammation induced by repetitive exposure to an aerosolized lysate of non-typeable *Haemophilus influenzae *(NTHi) [9]. NTHi is the most common bacterial colonizer of airways in COPD patients [10,11], present in the airways of 30% of COPD patients measured at a single point in time, and in more than 50% of COPD patients followed longitudinally [12]. We have shown that NTHi-induced COPD-like inflammation enhances lung tumorigenesis in a mouse model of lung cancer induced by airway epithelial expression of an oncogene (K-ras) [13]. Importantly, we and others have shown that there is a clear specificity for the nature of inflammation in lung cancer promotion, because induction of asthma-like (Th2 type) airway inflammation using weekly exposure to ovalbumin (OVA) aerosol in the same K-ras mutant mouse model [14] or in the urethane-induced lung cancer mouse model [15] did not result in a significant difference in lung surface tumor number.

Because tobacco smoking is a major cause of lung cancer, and smokers with COPD have the highest risk of lung cancer, it is also important to define how bacterial-induced COPD-like airway inflammation and tobacco carcinogen exposure alter lung tumorigenesis in mice. In the current study we have addressed this question using a tobacco-specific carcinogen exposure followed by an NTHi exposure in a newly developed genetic mouse model of lung cancer induced by lack of a tumor suppressor gene. This mouse model is based on the knockout of the retinoic acid inducible G protein coupled receptor *Gprc5a *gene, which leads to late-onset, low multiplicity lung tumor formation as previously described [16]. This differs substantially from the model we have used previously (the K-ras oncogene induced mouse model of lung cancer), and better emulates the typical course of lung cancer development in the setting of COPD in humans who have smoked cigarettes and become chronically colonized with bacteria. We found that Gprc5a-/- mice treated with NTHi alone develop chronic COPD-like inflammation and exhibit a several fold increase in the incidence of premalignant lesions. Furthermore, NTHi strongly enhanced lung tumor multiplicity in mice pretreated with the tobacco carcinogen 4-(methylnitrosamino)-1-(3-pyridyl)-1-butanone (NNK).

## Results

### Effect of NNK and NTHi on lung inflammation

**Figure 1:**
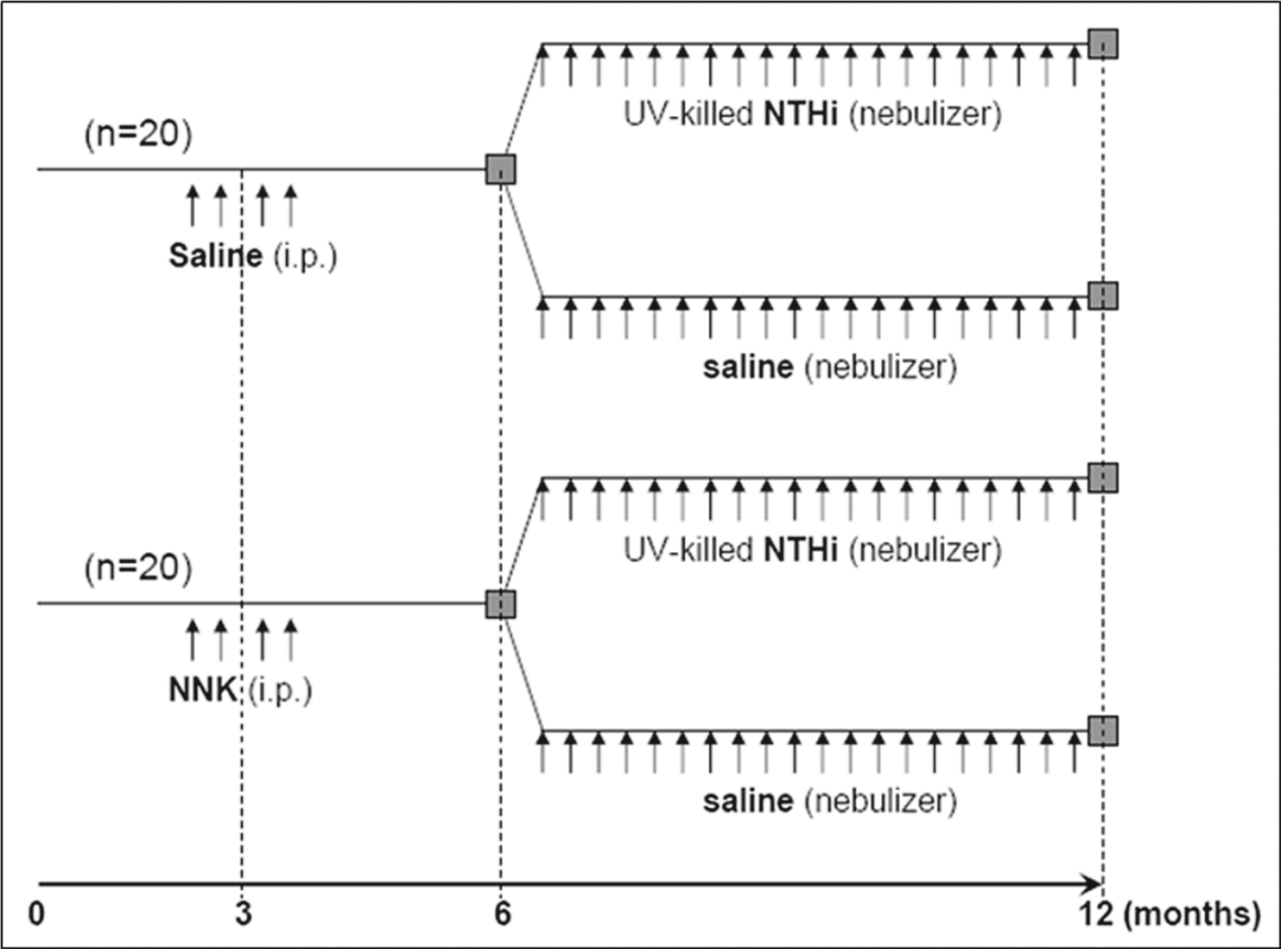
**Experimental design**. Gprc5a-/- mice were randomized to 4 consecutive, weekly injections of NNK or saline between the ages of 10 and 14 weeks, followed by exposure to an aerosolized UV-killed lysate of NTHi or saline between the ages of 6 and 12 months.

**Figure 2:**
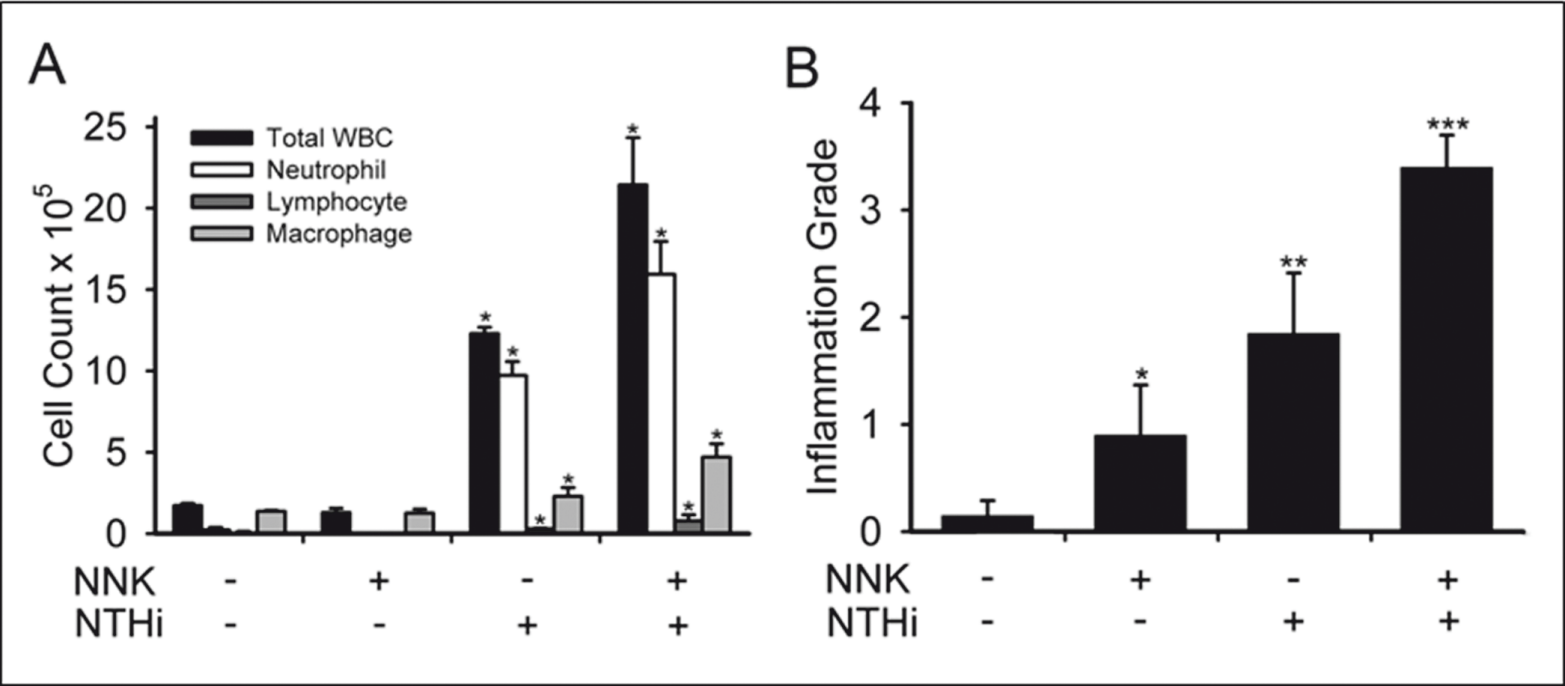
**Analysis of lung inflammation**. (A) Total and lineage-specific leukocyte numbers in bronchoalveolar lavage fluid (BALF) one day after the last NTHi or saline aerosol exposure are shown (n = 4, mean ± SE, *= P < 0.05 for NTHi or NNK/NTHi-exposed Gprc5a-/- mice vs PBS or NNK-exposed Gprc5a-/- mice). (B) Histologic grading of inflammation in lung tissue from each treatment group (n = 6, mean ± SE, *= P < 0.05 for NNK vs PBS, **= P < 0.05 for NTHi vs NNK, and ***= P < 0.05 for NNK/NTHi-exposed Gprc5a-/- mice vs NTHi-exposed Gprc5a-/- mice).

To test the role of airway inflammation in promotion of lung tumorigenesis, we exposed Gprc5a-/- mice to 4 weekly intraperitoneal (i.p.) injections of NNK from the age of 10 to 14 weeks, or to weekly NTHi lysate aerosol once weekly for 20 weeks starting at age 6 months, or to NNK followed by NTHi (Figure 1). Analysis of bronchoalveolar lavage fluid (BALF) collected from these mice showed that exposure to NNK alone resulted in a slight rise in the numbers of macrophages and neutrophils, while exposure to NTHi alone resulted in a marked influx of neutrophils, macrophages, and lymphocytes, with total leukocyte numbers increasing 7-fold (Figure 2A). Notably, the sequential exposure to NNK and NTHi increased even further the infiltration of inflammatory cells into the lung (Figure 2A). Histologic analysis of inflammation in lung tissue demonstrated that while the control group of Gprc5a-/- mice showed almost no inflammation, progressive increases in inflammation were observed in the groups treated with NNK only, NTHi only, and NNK followed by NTHi (Figure 2B).

Leukocyte recruitment in mice exposed to NNK, NTHi, or NNK/NTHi was accompanied by increases in cytokines and chemokines in BALF (Table 1). There were increased levels of proinflammatory cytokines (IL-6, TNF-α, and IL-1β), IFN-γ, IL-17 and neutrophil (KC, MIP-2), monocyte (MIP-1α), and CD8 cell (MIP-1α) chemokines. NNK alone and NTHi alone each increased the levels of certain cytokines and chemokines, but the greatest fold change in the level of most cytokines and chemokines was found in the dual NNK/NTHi-exposed group.

**Table 1:** Inflammatory mediators detected in BALF collected the day after the last treatment

Challenge	PBS	NNK	NNK:PBS	NTHi	NTHi:PBS	NNK+NTHi	NNK+NTHi:PBS
**Cytokines**							

**TNF-α**	13.4 ± 1.5	67.9 ± 9.5	5.1 *	59.4 ± 2.5	4.4 *	48.5 ± 5.2	3.6 *

**IL-1β**	4.1 ± 0.6	18.5 ± 1.2	4.5 *	21.2 ± 2.4	5.2 *	52.5 ± 4.9	12.8 *

**IL-6**	1.7 ± 0.4	4.1 ± 0.3	2.4 *	60.8 ± 17.5	35.7 *	128.3 ± 20.5	75.4 *

**IFN-γ**	8.8 ± 1.4	95.2 ± 11.7	10.8 *	59.1 ± 5.4	6.7 *	36.1 ± 3.2	4.1 *

**IL-17**	0.4 ± 0.1	1.1 ± 0.1	2.7 *	46.9 ± 1.9	117.2 *	19.9 ± 0.3	49.7 *

**Chemokines**							

**KC**	4.7 ± 1.3	8.8 ± 1.8	1.9	9.7 ± 0.4	2.1	70.9 ± 10.6	15.1 *

**MIP-1α**	1.3 ± 0.3	2.7 ± 1.1	2.1	7.6 ± 0.7	5.8 *	13.8 ± 2.6	10.6 *

**MIP-2**	12.9 ± 0.3	22.8 ± 6.8	1.7	19.3 ± 1.1	1.5 *	44.3 ± 1.7	3.4 *

Data are expressed in pg/ml and presented as the mean ± SEM (n = 3), and *= P < 0.05

BALF, bronchoalveolar lavage fluid; NNK, 4-(methylnitrosamino)-1-(3-pyridyl)-1-butanone; NTHi, non-typeable *Haemophilus influenzae*; TNF-α, tumor necrosis factor alpha; IL-1β, interleukin 1 beta; IL-6, interleukin 6; IFN-γ, interferon gamma; IL-17, interleukin 17; KC, keratinocyte-derived chemokine; MIP-1α, macrophage inflammatory protein 1 alpha; MIP-2, macrophage inflammatory protein 2.

### Effect of NNK and NTHi on lung tumor progression

**Figure 3:**
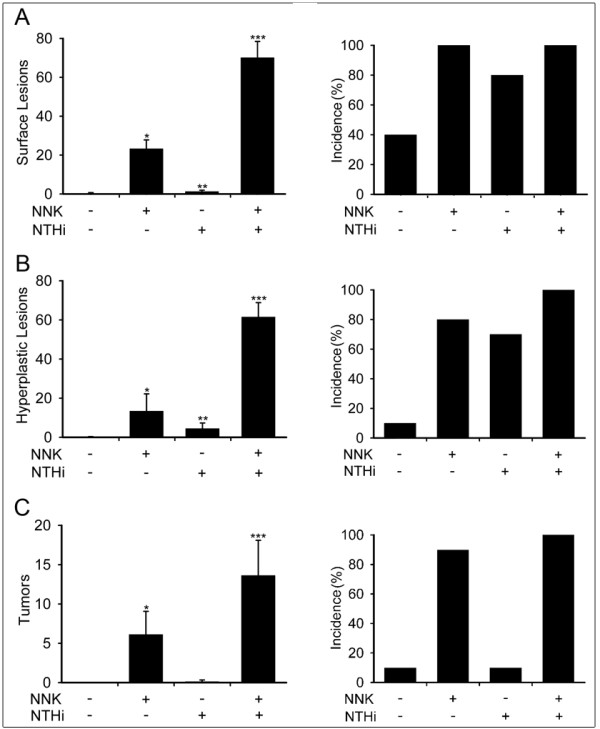
**Analysis of lung carcinogenesis**. (A) Lung surface lesion numbers (left) and incidence of lesion development (right) in Gprc5a-/- mice. (B) Number and incidence of hyperplastic lesions in the lungs of Gprc5a-/- mice. (C) Number and incidence of tumoral lesions in the lungs of Gprc5a-/- mice. (Incidence is calculated as the percentage of mice with surface lesions, hyperplastic lesions, or tumoral lesions among the total mice in the same study group, and presented as mean ± SE, *= P < 0.05 for NNK vs PBS, **= P < 0.05 for NTHi vs NNK, and ***= P < 0.05 for NNK/NTHi-exposed Gprc5a-/- mice vs NTHi-exposed Gprc5a-/- mice).

The effect of NNK and NTHi alone or in combination on lung tumor progression was analyzed by determining the number and histological features of lesions present on the pleural surface and in the parenchyma of the lungs of Gprc5a-/- mice. As shown in Figure 3A, NNK exposure lead to the development of multiple lung surface lesions (23.25 ± 4.5 in NNK-exposed vs 0.38 ± 0.29 in control mice) and increased epithelial hyperplasia (Figure 3B) and tumors (Figure 3C). In contrast, chronic NTHi exposure alone induced minor increases in lung surface lesions (Figure 3A) and tumors (Figure 3C), but it did cause an increase in proliferative lesions (Figure 3B). Mice exposed sequentially to NNK and NTHi showed a 3.5-fold increase in the multiplicity of surface lesions compared with mice exposed to NNK only (70.13 ± 8.28 vs 23.25 ± 4.5). Similarly, the multiplicity of hyperplastic lesions and tumors was higher in the NNK/NTHi than in the NNK only group by 4.5- and 2.2- fold, respectively (Figure 3B, and 3C). The incidence of surface lesions and hyperplastic lesions was increased in all three treatment groups compared to the untreated controls (Figure 3A and 3B), and the incidence of hyperplastic lesions in Gprc5a-/- mice exposed to both NNK and NTHi reached 100% (Figure 3B). However, NTHi alone did not increase the incidence of tumors even though the mice treated with both NNK and NTHi had a higher tumor incidence than mice exposed to NNK only (Figure 3C).

**Figure 4:**
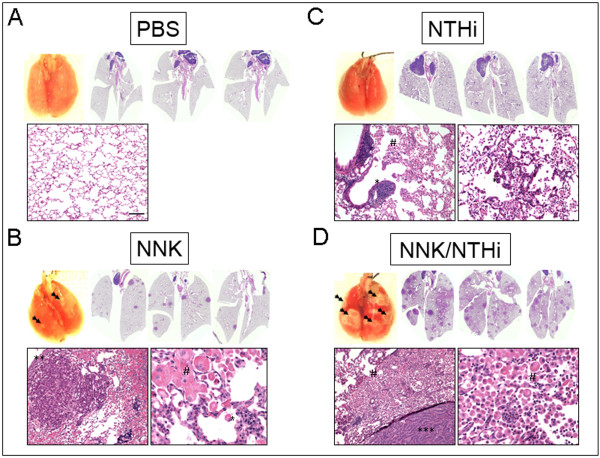
**Gross and microscopic analysis of lungs in Gprc5a-/- mice**. (A) Surface of freshly removed, formalin-filled control lungs of 12 months old Gprc5a-/- mice lacks visible lesions. Scans of H&E stained lung sections (2X) and microscopic image (10X) shows no lesion. (B) A few surface lesions (double arrows) were seen after NNK treatment. Scans of H&E stained cross sections of NNK-treated lung (2X) and high magnification (10X) microscopic images demonstrate the appearance of several papillary adenomas. (C) NTHi treatment led to perivascular and peribronchiolar lymphocyte infiltration (**) and characteristic alveolar accumulation of acidophilic macrophages, often resulting in confluent areas of acidophil macrophage pneumonia (AMP, #). AMP strongly co-localized with the widespread appearance of NTHi-induced premalignant, hyperplastic lesions (10X). (D) NNK+NTHi combined treatment led to confluent lesions on the lung surface (double arrows). A high number of pulmonary adenomas and areas of AMP are seen on the H&E scans (2X). Pulmonary hyperplasia with co-localized acidophilic macrophages (#) and papillary adenocarcinomas (***) with surrounding of heavy inflammatory infiltration characterized by the accumulation of macrophages and lymphocytes are seen on the microscopic images (10X, scale bar = 100 μm).

All tumors were classified according to morphology and there was a preponderance of papillary adenomas in all groups (the highest percentage seen in NTHi, and NTHi + NNK groups). Additional morphologic types in the NNK + NTHi group were observed including solid and mixed adenomas, and papillary adenocarcinoma, but their numbers were not significant. Representative macroscopic and microscopic histopathologic images of lung sections from each treatment group are shown in Figure 4. Histological examination revealed that the lungs of untreated Gprc5a -/- mice contained almost no tumor or hyperplastic lesion at the age of one year (Figure 4A). The lungs of Gprc5a -/- mice exposed to NNK alone showed an average of 13 foci of bronchiolar and/or alveolar hyperplasia, and an average of 2 adenomas (Figure 4B). These lesions were associated with macrophage infiltration (Figure 4B, lower right panel). The lungs of Gprc5a -/- mice exposed to NTHi alone showed an average of 5 foci of bronchiolar and/or alveolar hyperplasia and 1 adenoma (Figure 4C), with infiltration of neutrophils, macrophages and lymphocytes in the airways and alveoli. The average numbers of hyperplastic lesions and adenomas increased to 61, and 14, respectively, in the lungs of Gprc5a -/- mice exposed to both NNK and NTHi (Figure 4D). This was associated with infiltration of neutrophils, macrophages, and lymphocytes (Figure 4D, lower right panel). Lymphocytes were seen in nearly all treatment groups and the distribution varied from peri-tumor, intra-tumor, perivascular, and peribronchiolar. In some cases, the anatomic distribution of this follicular lymphoid hyperplasia was consistent with bronchus-associated lymphoid tissue (BALT), but clear association with one group could not be made. BALTs were randomly distributed along the small airways but were usually located between a bronchiole and an artery.

### Effect of NNK and NTHi on microvascular density and HIF-1α expression

**Figure 5:**
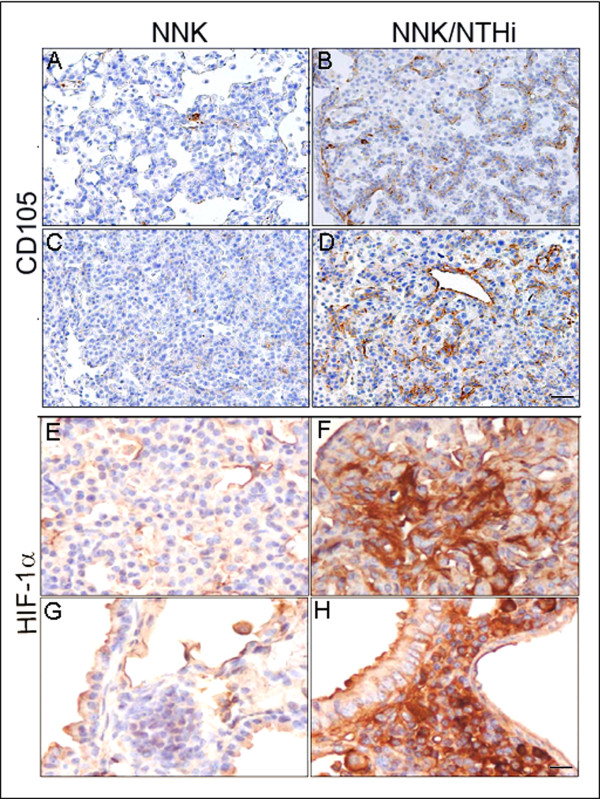
**Analysis of microvascular density and HIF-1α activity**. Microvascular density of pulmonary tumors was significantly higher in hyperplastic (B) and tumoral lesions (D) of NNK/NTHi treated mice compared to the NNK treated mice (A, and C) detected by CD105 immunostaining (10X, scale bar = 100 μm). HIF-1α immunostaining after NNK/NTHi combined treatment showed hot-spots of high stromal expression in tumors (F), and high expression in perivascular-peribronchiolar lymphocytes (H). In contrast, low, homogenous expression of HIF-1α was detected in the tumors (E) and perivascular-peribronchiolar lymphocytes (G) of NNK treated mice (40X, scale bar = 25 μm).

Because inflammation is often associated with angiogenesis [17] and angiogenesis is required for tumor promotion [18], we asked whether NNK and NTHi treatments affect microvascular density of lung lesions using the CD105 marker. Immunohistochemical staining of lung tissues showed enhanced CD105 expression within hyperplasia (Figure 5B) and tumors (Figure 5D) in NNK/NTHi-treated mice compared to NNK alone treated mice (Fig. A, and C), indicating significant increase in microvascular density of pulmonary tumors in response to NTHi. This was associated with hot-spots of high stromal hypoxia inducible factor 1alpha (HIF-1α) expression, an essential regulator of inflammation [19] and angiogenesis [18], in tumors (Figure 5F), and high expression in perivascular-peribronchiolar lymphocytes (Figure 5H), suggesting activation of HIF-1α related angiogenesis in response to NTHi-induced inflammation.

## Discussion

The likelihood of developing lung cancer within 10 years is 3-fold greater in patients with mild to moderate COPD and 10-fold greater in patients with severe COPD compared to smokers with normal lung function [4]. COPD is thought to be caused by the lung paranchymal response to inflammation from cigarette smoke and bacterial colonization of smoke-injured airways [10,20]. These epidemiologic data suggest that chronic airway inflammation caused by tobacco smoke and microbial infection promote lung carcinogenesis [21,22], and that lung cancer risk is positively associated with the severity and duration of inflammation [23]. However, while these data underlie the relationships, they do not establish causal links which are addressed in this study using a novel mouse model of lung carcinogenesis.

Tumor cells themselves produce various cytokines and chemokines that attract leukocytes (intrinsic inflammation). The inflammatory component of a developing neoplasm may include a diverse leukocyte population including neutrophils, dendritic cells, macrophages, and lymphocytes, all of which are capable of producing an array of cytokines, cytotoxic mediators including reactive oxygen species, serine and cysteine proteases, and matrix metalloproteinases (MMPs) [24,25]. Sustained cell proliferation in this environment rich in inflammatory cells, growth factors, activated stroma, and DNA-damage-promoting agents potentiates neoplastic risk [24,26]. We have previously shown that the loss of the tumor suppressor gene Gprc5a in mouse lung epithelial cells results in expression of various cytokines and chemokines, followed by recruitment of inflammatory cells into the lung tumor microenvironment [27]. Furthermore, increased activation of NF-κB in the Gprc5a-/- mouse lungs has been implicated in the creation of an inflammatory and proliferative microenvironment [27]. More recently, we have demonstrated that exposure of the Gprc5a knockout mice to the tobacco-specific carcinogen NNK enhances lung tumorigenesis [28]. In the present study, we have shown that extrinsic inflammation caused by bacterial infection and carcinogen exposure emulating COPD lung microenvironment, can further promote tumorigenesis in this mouse model. We previously showed the role of bacterial-induced COPD-like inflammation in promotion of lung tumorigenesis in an oncogenic K-ras induced mouse model of lung cancer [13]. Here we showed the role of bacterial-induced COPD-like inflammation in a background of tobacco carcinogen exposure in lung cancer promotion and progression in a mouse model with lack of Gprc5a activity. Deletion of Gprc5a, which is expressed preferentially in lung tissue, predisposes mice to develop spontaneous lung tumors [16], but the tumorigenesis process in the Gprc5a-/- mouse takes 1 to 2 years with low multiplicity. Even with exposure to NNK [28], the time to tumor development is slow (12 months). This is in contrast with the rapid development and high multiplicity of tumors in K-ras mutant mouse model [13]. Therefore, the Gprc5a knockout mouse model emulates better the typical course of lung cancer development in the setting of COPD in humans who have smoked cigarette and became chronically colonized with bacteria.

Cigarette smoke contains ~4,000 chemicals, of which ~60 have been identified as carcinogens [29]. Of these NNK is the most potent carcinogen in laboratory animals and has therefore been implicated as a significant cause of tobacco-associated cancers including lung cancer [29]. Activating mutations of K-ras, which are found in 30%-50% of lung adenocarcinoma (AC), are one of the most common genetic alterations associated with tobacco exposure [30]. Within days after NNK administration, K-ras becomes mutated and activated in alveolar type II pneumocytes and bronchiolar Clara cells, the putative cells from which lung AC originate [31,32]. However, in the present study, we showed that NNK exposure of Gprc5a knockout mice also results in increased levels of inflammatory mediators and recruitment of inflammatory cells into the lungs, which leads to lung tumor promotion. This is in agreement with the recent finding of a lung cancer promoting effect for smoke-induced inflammation independent of its direct mutagenesis effect in a K-ras mutant mouse model [33]. Chronic NTHi exposure in Gprc5a-/- mice resulted in increased levels of inflammatory mediators followed by recruitment of inflammatory cells into the lungs (COPD-like inflammation) and lung cancer promotion, similar to what we have previously described in our K-ras induced mouse model [13]. The highest levels of inflammation and tumor promotion were seen in Gprc5a-/- mice exposed to both NNK and NTHi, indicating an additive role for smoke- and bacterial-induced inflammation in progression from COPD to lung cancer.

Sustained angiogenesis is one of the hallmarks of cancer [34], and it is required for tumor promotion. We have also found increased microvessel density in response to NTHi-induced inflammation. This was associated with increased expression of HIF-1α which controls inflammation [19], and activates the transcription of genes involved in crucial aspects of cancer biology, including angiogenesis [18]. NF-κB is a critical transcriptional activator of HIF-1α activity and is required for HIF-1α protein accumulation during hypoxia [35-37]. Activation of NF-κB, a hallmark of inflammatory responses, is frequently detected in COPD patients [38], smokers [39] and tumors [40]. NF-κB is essential for promoting inflammation-associated cancers, and its inactivation decreases tumor multiplicity and delays cancer progression [41-43]. In the lung there are also a limited number of studies which show that NF-κB inhibition suppresses lung cancer in mouse [44,45]. We have previously found NF-κB activation in the lungs of wild type (WT) mice [9], Gprc5a-/- mice [27], and in mice with mutant K-ras exposed to NTHi [13,46]. We have also shown increased level of HIF-1α transcript in gene expression analysis of whole lung from WT, and mutant K-ras mice after NTHi exposure [14]. In this study, we found that a single exposure to aerosolized NTHi was sufficient to increase NF-κB activation five-fold in the lung of WT and Gprc5a-/- mice (data not shown), along with increased expression of HIF-1α (Figure 5). All together these data suggest a role for NF-κB in lung cancer promotion in the Gprc5a-/- mice in response to bacterial induced inflammation through activation of the HIF-1α pathway and its downstream angiogenic signals.

One of the events downstream of NF-κB activation is production of various cytokines and chemokines (e.g. IL-6, and IL-17) that attract leukocytes [41], which results in enhanced tumor progression, cancer cell growth and spread, angiogenesis, invasion and tumor immunosuppression [24,25]. We have found significantly increased level of IL-6 and IL-17 in BALF after NTHi exposure alone or in combination with NNK treatment (table 1). We have previously shown an essential role for IL-6 in promotion of lung cancer by airway inflammation [14]. IL-6 is required for differentiation of Th17 cells from naïve T cells, which mainly produce IL-17 [47,48]. IL-17 binds to the IL-17 receptor (IL-17R), and IL-17R signaling is required for lung CXC chemokine expression and neutrophil recruitment [49]. In addition to the traditional T helper 1 (Th1) response (IFN-γ) in COPD, recent developments in cytokine biology imply that COPD might be better explained by the Th17 phenotype [50,51]. These are consistent with our finding after inducing COPD-like inflammation by NTHi exposure (table 1 and Figure 2A). NNK exposure may induce a type of immune response (T regulatory response) which suppresses the anti-tumoral immune response (CD8 T cell, and Th1 responses) and meanwhile balances the Th17 response toward an effective pro-tumoral response.

During immune responses, neutrophils are among the first cells to arrive at sites of inflammation. This is similar to our finding of significant neutrophil recruitment after NTHi exposure in Gprc5a-/- mice (Figure 2A). An increased number of tumor-associated neutrophils (TANs) was linked to poorer outcome in patients with bronchioloalveolar carcinoma [52]. Neutrophils were present within the alveolar airspaces and within the tumor parenchyma during neoplastic development in a urethane-induced mouse model of lung cancer [53]. Using a K-ras induced mouse model, study has shown that TANs were involved in lung tumorigenesis by the production of elastase [54]. We have also shown an indirect anti-tumoral effect for curcumin through inhibition of neutrophil recruitment secondary to suppression of neutrophil chemoattractant (KC) in our K-ras induced lung cancer model [46].

## Conclusions

In conclusion, we propose that exposure of the airway to smoke and microbial products induces inflammation, which promotes the development of lung cancer. This is associated with activation of NF-κB, release of inflammatory mediators, recruitment of innate (neutrophil, and macrophages) and adaptive inflammatory cells, and activation of HIF-1α mediated angiogenesis. This will provide the basis for preclinical testing and rationally directed chemopreventive strategies to test the efficacy of anti-inflammatory and anti-bacterial agents targeting these signals in preventing carcinogenesis in patients at high risk for tumor development and early stage cancer.

## Methods

### Animals and treatment

*Gprc5a *knockout mice (Gprc5a-/- mice) were generated in a mixed background of 129sv × C57BL/6 as previously described [16]. The mice were maintained according to a protocol approved by the MD Anderson Cancer Center Institutional Animal Care and Use Committee in the specific pathogen-free animal facility, which is approved by the American Association for Accreditation of Laboratory Animal Care and is operated in accordance with current regulations and standards of the US Department of Agriculture and the Department of Health and Human Services. Mice were monitored daily for evidence of disease or death. Three-month-old Gprc5a-/- mice (n = 20) received 4 weekly i.p. injections of NNK (104 mg/kg of body weight) (Midwest Research Institute, Kansas City, MO) dissolved in saline solution (0.9% NaCl) or saline alone (n = 20), followed by weekly inhalation of the UV-killed lysate of NTHi (n = 10) or saline (n = 10) from the age of 6 to12 months (Figure 1). Briefly, a lysate of NTHi strain 12 was prepared as previously described [9]. The protein concentration was adjusted to 2.5 mg/ml in phosphate buffered saline (PBS), and the lysate was frozen in 10 ml aliquots at -80°C. To deliver the lysate to mice by aerosol, a thawed aliquot was placed in an AeroMist CA-209 nebulizer (CIS-US, Bedford, MA) driven by 10 l/min of room air supplemented with 5% CO_2 _for 20 minutes.

### Assessment of lung tumor burden and inflammation

One day after the last NTHi exposure, animals were euthanized by i.p. injection of a lethal dose of avertin (Sigma-Aldrich, St. Louis, MO). Lung surface lesion numbers were counted (any macroscopic lesion, tumoral or non-tumoral, seen with the naked eye on the lung surface), then the lungs were prepared for histological analysis as described below. In some mice (n = 5), BALF was obtained by sequentially instilling and collecting two aliquots of 1 ml PBS through a tracheostomy cannula. Total leukocyte count was determined using a hemacytometer, and cell populations were determined after cytocentrifugation of 300 μl of BALF followed by Wright-Giemsa staining. The remaining BALF (~1.4 ml) was centrifuged at 1,250 × *g *for 10 min, and supernatants were collected and stored at -70°C. Cytokine concentrations were measured in duplicate by multiplexed sandwich ELISA using SearchLight Proteome Arrays (Aushon Biosystems, Billerica, MA).

### Histochemistry

The tracheas of euthanized mice were cannulated with PE-50 tubing and sutured into place. The lungs were perfused in situ with PBS via the right cardiac ventricle, then infused with 10% buffered formalin (Sigma-Aldrich), removed and placed in 10% buffered formalin for 18 h. Tissues then were transferred to 75% ethanol, embedded in paraffin and sectioned at 5-μm thickness. Sections were dried at 60°C for 15 min, and then were deparaffinized. Hematoxylin and eosin (H&E) staining was performed by incubating the tissues in Harris hematoxylin (Sigma-Aldrich) followed by serial eosin and graded ethanol steps. Three H&E sections, each 600 μm apart, were analyzed blindly by a veterinary pathologist for proliferative lesions, tumor (adenoma + adenocarcinoma) multiplicity, incidence, inflammatory infiltrations and overall inflammation grade according to the recommendations of the Mouse Models of Human Cancer Consortium [55]. The severity of inflammatory lesions of the lungs were scored from 1 to 4 as follows: grade 1 - minimal, lesions affect less than 10% of tissue; grade 2 - mild, lesions affect 10-20% of tissue; grade 3 - moderate, lesions affect 21-40% of tissue; and grade 4 - marked or severe, lesions affect 41-100% of the tissue. Some sections (5 μm) from NNK- and NNK/NTHi-treated groups were labeled with anti-CD105 antibody (Biocare Medical LLC., Concord, CA) after antigen retrieval procedure for 20 min at 95°C in Tris-EDTA buffer, pH 9.0, and HIF-1α antibody (Novus Biologicals, Littleton, CO, USA). Slides were then incubated with secondary antibody, exposed to horseradish peroxidase-labeled streptavidin (Vector, Burlingame, CA), developed with diaminobenzidine (Vector, Burlingame, CA), and counterstained with hematoxylin (Sigma-Aldrich).

### Statistical methods

Summary statistics for cell counts in BALF and tumor counts were computed within treatment groups, and analysis of variance with adjustment for multiple comparisons was performed to examine the differences between the mean cell counts and tumor count of the control group and each of the NTHi and or NTHi/NNK treatment groups. Differences were considered significant for P < 0.05.

## List of abbreviations

COPD: chronic obstructive pulmonary disease; NTHi: non-typeable *Haemophilus influenzae; *NNK: 4-(methylnitrosamino)-1-(3-pyridyl)-1-butanone; PBS: phosphate buffered saline; BALF: bronchoalveolar lavage fluid; H&E: hematoxylin and eosin; AC: adenocarcinoma; TANs: tumor-associated neutrophils: NF-κB: nuclear factor kappa B; HIF-1α: hypoxia inducible factor 1α.

## Competing interests

The authors declare that they have no competing interests.

## Authors' contributions

PB carried out the mouse *in vivo *study including NNK injection, weekly NTHi exposure, and lung tissue extraction, and participated in preparing the figures and drafting the manuscript. CVP carried out the histopathology examination and analysis of the lung tissues and participated in preparing the figures. TM participated in the mouse colony maintenance, genotyping, and lung tissue extraction. BFD participated in the design of the study and the drafting the manuscript. RL conceived of the study, and participated in its design and coordination and helped to draft the manuscript. SJM participated in the design of the study, assessed lung tumor burden and inflammation, performed the statistical analysis, and participated in preparing the figures and drafting the manuscript. All authors read and approved the final manuscript.
